# Decoding China’s COVID‐19 ‘virus exceptionalism’: Community‐based digital contact tracing in Wuhan

**DOI:** 10.1111/radm.12464

**Published:** 2021-03-23

**Authors:** Philipp Boeing, Yihan Wang

**Affiliations:** ^1^ Department of Economics of Innovation and Industrial Dynamics ZEW – Leibniz Centre for European Economic Research L 7, 1 Mannheim 68161 Germany; ^2^ Department of Strategy and Entrepreneurship EM Normandie Business School Métis Lab, 20 Quai Frissard Le Havre 76600 France

## Abstract

During the COVID‐19 pandemic, comprehensive, accurate, and timely digital contact tracing serves as a decisive measure in curbing viral transmission. Such a strategy integrates corporate innovation, government decision‐making, citizen participation, and community coordination with big data analytics. This article explores how key stakeholders in an open innovation ecosystem interact within the digital context to overcome challenges to public health and socio‐economic welfare imposed by the pandemic. To enhance the digital contact tracing effectiveness, communities are deployed to moderate the interactions between government, enterprises and citizens. As an example, we study the community‐based digital contact tracing in Wuhan, a representative case of China’s ‘virus exceptionalism’ in COVID‐19 mitigation. We discuss the effectiveness of this strategy and raise critical ethical concerns regarding decision‐making in R&D management.

## 1 Introduction

The COVID‐19 pandemic imposes enormous challenges to public health and socioeconomic welfare. The crisis necessitates technological and managerial innovation for business strategy, public administration, and government policymaking in curbing the viral spread and simultaneously maintaining economic activity and social life (Verma and Gustafsson, 2020). The high transmissibility, long incubation period, and existence of asymptomatic patients not only expose the ineffectiveness of traditional mitigation techniques (e.g., wearing masks, keeping physical distance, restricting long‐distance travels) but also lead to a rapid exhaustion of public health services (Nguyen et al., 2020). Meanwhile, due to the long duration and uncertainty in vaccine development and implementation, timely and accurate contact tracing is urged as a prioritized response to prevent transmission and spread (Kwok et al., 2019; Kretzschmar et al., 2020). Notwithstanding, timely manual contact tracing of infected cases and their close contacts are largely constrained by the available personnel deployed in the process, thus cannot sufficiently monitor human‐to‐human transmission once new infections reach a certain level.

Composed of two criteria, completeness (the exhaustive coverage of public health data) and timeliness (the immediate identification and reaction to outbreaks), effective digital contact tracing incorporates latest information and communications technologies (ICT) to enhance the tracing measures and coordinate communication at the societal level (Kretzschmar et al., 2020). Prior medical and epidemiological research mostly sees the implementation of digital contact tracing as a top‐down government intervention (e.g., Kwok et al., 2019; Ferretti et al., 2020; He, 2020; Kretzschmar et al., 2020; Nguyen et al., 2020; Pan et al., 2020). However, the mechanisms that facilitate the interactions among key stakeholders in digital contact tracing are not sufficiently addressed in prior studies.

In this regard, we explore how the interactions among key stakeholders (enterprises, government, citizens, and communities) in an open innovation ecosystem contribute to effective digital contact tracing. The open innovation ecosystem is a wide range of social forces that integrate the full spectrum of competitive and cooperative relationships engaged in such processes (Chesbrough and Bogers, 2014; Sklyar et al., 2019). More precisely, it engages multiple key stakeholders on three ‘grounds’ – the ‘upperground’ organizations (e.g., enterprises and government), the ‘middleground’ communities (e.g., public collectives, associations, and trade unions), and the ‘underground’ individuals (e.g., citizens) (Bovaird, 2007; Cohendet et al., 2010; Grandadam et al., 2013; Gustafsson and Jarvenpaa, 2018). Embedded in an open innovation ecosystem, digital contact tracing embodies the big data analytics of public health information, which depends on four key components – pooled internal and external data sources generated by the individual citizens, data transformation processes facilitated by the enterprises, tools of data analysis and sharing platforms moderated by the communities, and data application outputs by the government (Raghupathi and Raghupathi, 2014).

In this article, we explore the social conditions that facilitate the effectiveness of digital contact tracing based on a chronological case study of the outbreak and subsequent control measures of COVID‐19 in Wuhan, China. As the world’s ground zero of COVID‐19, China was broadly criticized for its initial mismanagement to control the viral spread. However, less attention has been paid to China’s ‘virus exceptionalism’ that introduces harsh measures (e.g., draconian lockdown of megacities, digital contact tracing systems, and mandatory quarantine upon arrival for international travelers) to achieve a viral reproduction rate (*R*_0_)^1^ close to zero in a short period, as well as maintain economic growth^2^ regardless of the pandemic (*The Economist*, April 30, 2020). The case study of Wuhan exhibits how digital contact tracing integrates the actions of key stakeholders and the important roles of middleground communities in an open innovation ecosystem. Meanwhile, we reiterate the critical ethical trade‐offs of the community‐based digital contact tracing in privacy protection, information transparency, and social justice concerning its effectiveness (Angst and Agarwal, 2009; Clarke and Margetts, 2014; Parker et al., 2020). The discourse analysis is grounded on unique ethnographic observations in Wuhan, and secondary sources from Chinese government press releases, international media coverage, seminal epidemiological publications, and embassy reports.

## 2 Conceptual background

### 2.1 Digital contact tracing and open innovation ecosystem

The constantly evolving COVID‐19 pandemic involves multidisciplinary research that contributes to a roadmap for business strategy and public policy (Verma and Gustafsson, 2020). Recent statistics of OECD countries show that the relationship between protecting people’s health and maintaining economic growth is not a ‘trade‐off’ but rather goes hand‐in‐hand during the COVID‐19 pandemic (Hasell, 2020). Leveraging the full benefits of digital technology, a well‐functioning open innovation system contributes to both public health and socioeconomic well‐being (Greve, 2015; Schmidthuber et al., 2019).

Based on the big data analytics of first‐hand public health information, digital contact tracing provides a scalable and effective solution to detect and monitor the spread of viral diseases and reduce the risk of human‐to‐human transmission (Hao et al., 2020). Furthermore, predictive analytics of digital contact tracing allows for the early identification of future risk scenarios and required countermeasures, which also allow for more targeted intervention and quarantine. As a result, the government can deliver timely public services to citizens according to different risk levels, while simultaneously supporting public health as well as economic activities. Several pilot medical research has confirmed the effectiveness of digital contact tracing and argue that digital solutions sharply contain the spread of COVID‐19 by shortening the testing delay and enclosing the infection pathway^3^ (Braithwaite et al., 2020; Ferretti et al., 2020; He, 2020; Kretzschmar et al., 2020). Nevertheless, without strong enforcement and coordination mechanisms at the societal level, only digital technology alone cannot sufficiently complement traditional mitigation measures for contact tracing effectiveness.

We argue that the effectiveness of digital contact tracing requires proactive interactions among key stakeholders in an open innovation ecosystem including enterprises, government, citizens, and communities. First and foremost, the open innovation ecosystem entails the interaction between corporate innovation activity and government public administration. As the main technological facilitator, enterprises are urged to open up their innovation processes to absorb external knowledge and diffuse in‐house expertise in coordinating various value‐creating activities with diverse social players (Chesbrough et al., 2006). As the orchestrator of digital contact tracing measures, the government legitimizes and authorizes corporate innovation activities in sensitive areas, essentially the collection and analysis of citizens’ personal information, to create ‘public value’ through public–private collaboration (Greve, 2015). Under government endorsement, enterprises develop and maintain the operations of digital contact tracing platforms as well as provide technological support in analytics of public health data. In this process, enterprises absorb, incubate, and diffuse new technological and market knowledge that propels innovation activities and business model upgrading (Chesbrough et al., 2006; Wang and Turkina, 2020; Spieth et al., 2021). Reciprocally, by collaborating with the private sector, the government utilizes the outcomes of public health data analytics for real‐time decision making and deliver public services on the digital platform. Subsequently, the government’s embeddedness in the open innovation networks enhances public incentives for innovation in areas where market incentives are insufficient (Levén et al., 2014; Boeing, 2016; Wang et al., 2018).

Additionally, large‐scale citizen participation plays an indispensable role that contributes to the timeliness and accuracy of digital contact tracing. From the perspectives of the enterprises, broad citizen participation via smartphone apps provides a large mass of real‐time personalized data as strategic resources. In return, the shared user’s experience and feedback improve their product and service quality. From the perspectives of the government, digital contact tracing advocates the reconciliation of the common interests in digital‐era governance (transparency on public digital platforms), public value management (innovation‐driven strategy‐making and performance governance), and collaborative governance (networks and collaborations in public–private partnership and engagement) (Greve, 2015). On the digital platforms of open innovation ecosystem, citizens provide personalized ‘open data’ in a consolidated format, so that the government can make timely decisions based on the big data analytics of ICT firms (Clarke and Margetts, 2014). Thereafter, the outputs of open innovation are accountable for the collective and individual interest of the public (Brown and Toze, 2017). On the principle of transparency, citizens can be motivated to participate in open innovation. New ideas and knowledge generated in citizen participation improve the government decision‐making effectiveness and public service quality (Chun et al., 2010; Schmidthuber et al., 2019).

### 2.2 Communities in the open innovation ecosystem

**Figure 1:**
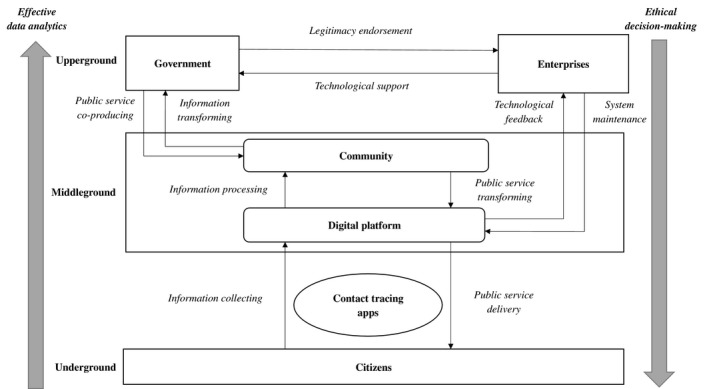
Community‐based digital contact tracing in the open innovation ecosystem.

Effective contact tracing integrates public need‐oriented government administration and policymaking, corporate technological innovation and business model upgrading, and broad engagement citizen participation in an open innovation ecosystem (Mulgan, 2006). The social exchanges among them are often not directly performed bilaterally, but rather moderated on the intermediate interface of middleground communities between upperground organizations and underground individuals. Figure 1 presents the general framework of the interactions of the three ‘grounds’ of the open innovation ecosystem in the process of digital contact tracing.

The communities are collective social units of people ‘with diverse characteristics that are linked by social ties, share common perspectives, and engage in joint action in geographical locations or settings’ (MacQueen et al., 2001). In the administrative hierarchy, the communities serve as the physical intermediaries that co‐produce policy with the government and deliver services to the public (Bovaird, 2007). They represent the collective interests of affiliated citizens by creating public value, while autonomously moderate multilayer interactions and knowledge transmissions (Gustafsson and Jarvenpaa, 2018). Existing literature suggests how different forms of communities mitigate the multilayer interactions in the open innovation ecosystem (e.g., Local Emergency Planning Committees (LEPCs) in US residential communities in alerting natural disasters and terrorist attacks (McEntire David and Myers, 2004), Internet Engineering Task Force (IETF) in innovation project leaders selection and networking of engineers working groups (Fleming and Waguespack, 2007), and the video game community building in the creative city of Montréal (Grandadam et al., 2013)). In these examples, the community formation is led by the upperground organizations based on geographically and relational proximity and/or professional connections. By generating and leveraging the influence of communities on the digital platform, policymakers mobilize the collective intelligence and engagement of autonomous individuals to achieve the strategic goals in open innovation.

In digital contact tracing, the communities aggregate and transfer the first‐hand individual health data to the analytical functions of endorsed ICT firms, which later on present the consolidated outcomes to the government policymakers (Eckman et al., 2007). In return, the communities receive policy mandates and guidelines on the same platform from the government, who then defuse the content to affiliated members, including alert of the outbreak in the community, testing guidelines at community health centers, as well as the instructions of neighborhoods and daily necessities delivery services. When an epidemic breaks out, communities are both the first responders to citizens’ requests at the organizational level as well as the implementing agency of governmental commands at the public administration baselines (McEntire David and Myers, 2004). The citizens receive pertinent and practical information from their affiliated communities via the digital platform. Reversely, the increasing willingness of the citizens to participate in contact tracing enhances the effectiveness of digitized public administration (Angst and Agarwal, 2009; Schmidthuber et al., 2019).

### 2.3 Ethical concerns of digital contact tracing

Regardless of the necessity of containing the viral spread, digital contact tracing equivalently raises crucial ethical concerns for its purposes and means. The actual value of personal data lies in its contextualization with data points of the population. Successful big data analytics of digital contact tracing requires a large quantity of well‐processed and pooled data as well as responsive feedback that meet participants’ needs based on the trust of the public administration's credibility (Raghupathi and Raghupathi, 2014). However, digital technology will reach maximum effectiveness only if the near‐population uses the same app and is willing (or obliged) to share the generated data (Clarke and Margetts, 2014). Stating ‘the mere existence of an emergency does not in itself legitimize any intrusion on the autonomy or privacy of individuals or group’, Parker et al. (2020) reviewed several crucial ethical issues in the implementation of digital contact tracing, including privacy protection, freedom of choice, the responsibilities of institutions and professionals as well as social justice and fairness. In the short‐ to medium‐run, risks may include the misuse of data and technology or prolonged surveillance without consensus among citizens. In times of acute emergencies, there may be a larger collective willingness to compromise privacy for gains in security. However, in the long run, government and tech giants can monopolize the usage of public data collected in contact tracing as a ‘new normal’, which may infringe citizens’ right of choice and discretion even after the pandemic.

In this ethical dilemma, the community plays the intermediary role of leveraging the voices and power between underground individuals and upperground organizations. The benign interactions between the citizens and the government are grounded on public trust and confidence, which necessitate transparency and accountability from public administration (Clarke and Margetts, 2014). If the underground individuals are unwilling to provide their data to the upperground organizations, or the latter have the insufficient capability for real‐time data aggregation and analytics, which would adversely affect all stakeholders. Thereafter, the communities are designated to monitor the upperground organizations not only to ‘do things well’, but also ‘do things right’ – representing social justice and in public service co‐production and the common interest of the affiliated citizens.

## 3 Community‐based digital contact tracing in Wuhan

**Figure 2:**
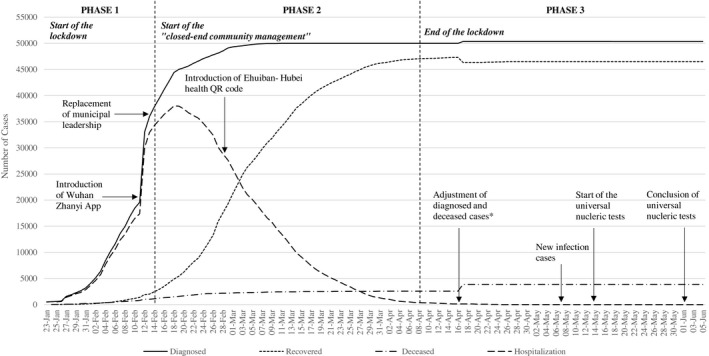
Covid‐19 daily cumulative diagnosed, recovered and deceased cases and remaining hospitalization in Wuhan. *Note*: On April 17 2020 the Health Commission of Wuhan announced an adjustment of increases in diagnosed and deceased cases, including unreported early cases due to previously limited testing and accommodation capacity. *Source*: National Health Commission of the People’s Republic of China, Health Commission of Hubei Province.

To illustrate how effective digital contact tracing engages key stakeholders in containing COVID‐19, we interpret a chronological case study of the digital contact tracing applied in Wuhan, China over three stages (see Figure 2). The evidence combines unique ethnographic observations by one of the authors in Wuhan from mid‐January to early June 2020 with secondary sources^4^ of press releases from the Chinese government, international media coverage, seminal epidemiological publications, and nonpublic status reports by the German Embassy and Consulates in China.^5^

### 3.1 Phase I: The emergence of COVID‐19 in Wuhan

It is widely acknowledged that in December 2019 the first known COVID‐19 patient was identified in Wuhan, the capital of Hubei Province and a major transportation hub in central China (Huang et al., 2020). Before January 20,^6^ the local authorities of Wuhan claimed no evidence of a human‐to‐human transmission phenomenon nor infections among medical staff. The public was provided little information on local infection risks or necessary preventive measures to take after spotting infection symptoms. Residents were still allowed to circulate within or depart from the city for the Lunar New Year holiday. Consequently, the infections not only increased locally but also expanded across the country and abroad (Chen and Yu, 2020). However, on January 20, a public health expert team dispatched from the central government of China to Wuhan fully overturned prior conclusions by the local authorities on low human‐to‐human transmission and also confirmed infections in hospitals. On January 23, the municipal government of Wuhan launched a draconian lockdown that suspended all local and external transportation connections as well as closed down almost all nonessential businesses.

**Figure 3:**
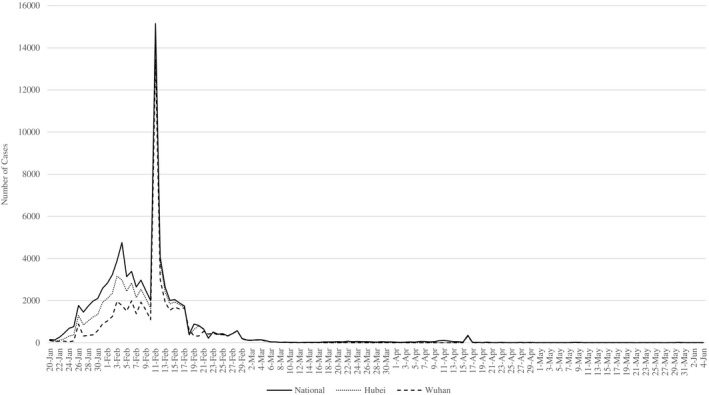
National, provincial and municipal daily increase in Covid‐19 diagnosed infections. *Source*: National Health Commission of the People’s Republic of China, Health Commission of Hubei Province.

To reduce the viral spread in public, the municipal government of Wuhan banned all unauthorized public and private transportation and urged residents to avoid unnecessary transfers in and out of the city. However, due to lacking location‐specific information on infections and coordinated crisis management, the scale of infections still escalated sharply (see Figure 3). Although some early interventions were undertaken, the infection rates still surged rapidly due to the shortage of personal protective equipment (PPE), the negligence of family‐based infection clusters, and delayed diagnosis and treatment (Pan et al., 2020). Because of numerous incoming patients, local healthcare resources and staff were immediately exhausted. Throughout this early mismanagement, the local authorities of Wuhan did not sufficiently pay attention to the potential of digital technology in providing authoritative and transparent public health information as well as mobilizing grassroot social forces in mitigating the viral spread. The public went through mass panic‐like uncertainty and searched for information from unauthorized social media sources, which further deteriorated the credibility of government decision making. The low effectiveness in curbing the viral spread and the increasing public dissatisfaction with the local government led to the replacement of local leadership boards of Hubei Province and Wuhan Municipality by the Chinese central government on February 13.

### 3.2 Phase II: Community‐based digital contact tracing during the lockdown

To overturn the disastrous situation in Phase I, the new leadership board of Wuhan swiftly introduced the ‘closed‐end community management’ strategy including a community‐based digital contract tracing mission in mid‐February. During the lockdown period, physical barriers were settled at the borders of residential neighborhoods to minimize physical movement. At the same time, residents were divided into community grids based on these boundaries as the basic unit of digital contact tracing.

The community‐based digital contact tracing combined the digital technological development with the social interactions in an open innovation ecosystem in community administration. Endorsed by the government, ICT firms first developed contact tracing apps implanted on ubiquitously used communication and online payment digital platforms, such WeChat (Tencent) and Alipay (Alibaba),^7^ to enable the big data analysis. In Wuhan, two contact tracing apps were introduced at the municipal and provincial level (see Table 1), namely, Wuhan Zhanyi on the WeChat platform and Ehuiban‐Hubei Health QR Code on the Alipay platform (see Table 1). On both apps, individual users were requested to provide the personal information (e.g., ID number, demographic information, verified cellphone number, domicile address, and family member information), health conditions (body temperature, possible infection symptoms, and contact with diagnosed or suspected patients), and travel history before the lockdown. Then, the digital contact tracing app automatically generated a health QR code as the digital ID and assign a community grid for monitoring.

**Table 1:** Community‐based contact tracing apps in Wuhan

Contact tracing App	Wuhan Zhanyi (武汉战疫)	Ehuiban‐Hubei Health QR code (鄂汇办‐湖北健康码)
Icon	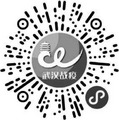	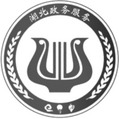
Authorization	Wuhan Government Service and Big Data Administration Bureau	Hubei Provincial Government Transparency and Services Administration Office
Date of introduction	February 9, 2020	February 29, 2020
Administrative level	Municipality	Province
Digital platform (number of active users in China, March 2020)	WeChat (983.18 million)	Alipay (698.56 million)
Supporting ICT firm (annual revenue in 2019)	Tencent Holdings Ltd. (52.82 billion USD)	Alibaba Group (52.76 billion USD)
Main functions	Acquire electronic permission to enter residential neighborhoods and public places in the city of Wuhan. Consult information on real‐time epidemic statistics, hospital vacancies in the municipality, and volume of nearby population flows	Acquire electronic permission to travel and work within the territory of Hubei Province. Report information on possible infections in the province. Consult information on hospital vacancies in the province, and policy on epidemic prevention and control

Meanwhile, civil servants and volunteers were dispatched as contact tracers (or grid correspondents) in the community grids to get in contact with residents. Their main tasks included conducting door‐to‐door surveys on household health conditions, providing information on public services, coordinating daily essential goods supplies, and contacting emergency service once local infections are identified. At the physical entrance of the closed‐end communities, contact tracers were stationed to check the ID information and health condition by scanning their health QR codes presented on the tracing apps. Since downloading the contact tracing app and generating personalized health QR codes were the prerequisites of physical mobility outside the closed‐end community, these monitoring measures ensured the broad user coverage of contact tracing apps downloads at the individual level. Moreover, the community contact tracers also facilitated the medical services on the digital platform during the lockdown period. Since all public and private transportation means were banned, people with COVID‐19 symptoms could only arrange transportation to the next available hospital vacancies or quarantine centers by contacting the contact tracers. While coordinating patient transportation, the community contact tracers further aggregated the local outbreak information in the big data analytics system. The government and supporting ICT firms compared aggregated community‐level information with administrative databases to estimate the risk of infection within local communities, and then identify closed contacts of diagnosed cases for quarantine. Based on the real‐time contact tracing data, the municipal government could effectively track potential viral spreading paths. Moreover, the government could assess community‐level spreading risk and adopt differentiated measures to lift the lockdown. The more complete and accurate digital contact tracing with differentiated lockdown measures contributed to the sharp drop in new infections and an increase in hospital discharge, which laid down the evidential ground for lifting the lockdown.

### 3.3 Phase III: Evolving contact tracing measures after the lockdown

After 76 days of draconian lockdown, on April 8, the municipal government lifted physical restrictions. Meanwhile, many digitalized contact tracing measures are still maintained to prevent a ‘second wave’ and the mission of community‐based digital contact tracing is shifted from detection toward prevention.

First and foremost, the digital health QR code turned into colored electronic permission for individuals to return to work and travel. Based on the tracing data of local infections collected during the lockdown period, the government and ICT firms divided all local community grids into three risk levels – high, medium, and low on a dynamic basis. On the ground of these three tiers, residents were assigned colored health QR codes on the contact tracing app terminals based on the risk of community infection. First, the diagnosed and suspected infecting patients together with their close contacts were assigned ‘red codes’, which required 14‐day quarantine at home or at the quarantine centers. The residents in the same community grid of the red code assignees would be assigned ‘yellow codes’ for a 7‐day quarantine at home. The prior mobility tracks of red and yellow code holders would be immediately reported to the local health authorities and released to the public on the digital platforms. Based on the big data analytics of red and yellow code holder clusters, the municipal government publicly announced the list of high or medium risk communities and deployed dynamic ‘closed‐end management’ within these communities accordingly. Otherwise, a community grid would be announced as a ‘non‐infection community’, if no red code or yellow code holders were detected for 14 consecutive days. The affiliated members were assigned ‘green codes’, which allowed full access to public places and transportation as well as the permission to return to work. To ensure the effectiveness of this dynamic controlling measure, the contact tracers checked the health QR code colors and registered the body temperature of passersby at the entrance of residential areas, public places, or public transportation. These measures ensure tight monitoring of human mobility and timely identification of close contacts in public places once a positive case is diagnosed.

To further eradicate threats of a ‘second wave’, on May 12 the municipal government of Wuhan launched an ambitious plan of conducting universal nucleic tests of all residents. Once again, the communities were deployed as the intermediary communication and coordination channel between the government and the citizens. The municipal government established temporary testing centers in the public places of residential neighborhoods. Trained medical professionals were dispatched to these centers to conduct swab and/or blood tests. The contact tracers continuously utilized the contact tracing apps to inform residents about assigned time slots and test locations. When entering the testing centers, residents were requested to scan their health QR codes and radio‐frequency identification (RFID) ID cards to generate a personalized barcode tagging on the testing samples of each participant. Since the personal information was already consolidated during the health QR code registration, the registration procedure was largely simplified, so that the residents could immediately take the test and leave the center with minimum human contact. Via the contact tracing apps, residents with limited mobility, and those who missed the grouped testing could also request individualized testing at home. Within 1 week, the residents automatically received their nucleic testing results on the contact tracing apps to determine if they were permitted to return to work.

On June 2, the provincial government of Hubei announced that from May 14 to June 1, the universal nucleic tests covered 9,899,828 residents. In total 300 asymptotic cases were identified, and 1,174 close contacts were traced and underwent quarantine. After that date, no local infections have been reported and the city of Wuhan reintroduced no lockdown measures till the end of 2020.

## 4 Case interpretation

### 4.1 Effectiveness of big data analytics

The practice of community‐based digital contact tracing in Wuhan appears to demonstrate how digital technology engages multiple key stakeholders in an open innovation ecosystem in crisis. As the world’s first megacity hit by COVID‐19, Wuhan witnessed a disastrous initial stage of mismanagement. Initial disregard for digital technology and community engagement resulted in the rapid exhaustion of public health resources and panic among citizens. Later on, the engagement of key stakeholders, specifically the middleground communities, in an open innovation ecosystem not only increases the completeness and timeliness of digital contact tracing to curb the viral spread but also contribute to the re‐establishment of economic activity and social life.

In the upperground, the government’s endorsement and collaboration with private firms such as Tencent and Alibaba was an important foundation for rapid public–private collaboration. Technologically supported by ICT firms, the municipal government became capable of conducting big data analytics that aggregated citizens’ health and geolocation information via the contact tracing platform. Thereafter, the government could undertake timely intervention measures and provide urgently required public services. In turn, the ICT firms that successfully bid the right to develop contact tracing apps and platforms gained legitimacy in crisis management and extended their base of public or private customers.

In the middleground, the communities co‐produced public services and coordinated communication with individuals on the digital platform. On the one hand, the communities aggregated real‐time data clusters for more complete and accurate big data analytics at the upperground. On the other hand, the communities implimented the monitoring measures and provided personalized services. They also ensured the broad underground citizen participation and intervened in cluster infections together with upperground organizations. Finally, the communities served as the social force to ensure the smooth transition in lifting the lockdown including controlling health QR codes for returning workers and coordinating universal nucleic tests.

In the underground, the essential contact tracing systems were built on ubitiously used digital platforms, that is, WeChat and Alipay, so that the citizens encountered little operational hurdles to get adapted to the system. Individual users constantly provide first‐hand public health and geospatial data through contact tracing apps installed on their smartphones. In return, they received timely analytical results of the detected community transmission as well as the public service. Thanks to the broad coverage in the underground and timely reacted contact tracing forwarded by the middleground communities and digital platform, the upperground organizations could improve the accuracy of big data analytics of public health information, and then implement differentiated preventive measures on communities of different risk levels.

In all, the community‐based digital contact tracing in Wuhan combines the digital technological advances and the mobilization of multiple social forces embedded in the open innovation system. Regardless of the initial mismanagement, Wuhan established a digital contact tracing system that operates at significantly lower socioeconomic costs than what repeated lockdowns may incur, which provides a safeguard for the citizens to return to work. Reconstructing the full transmission dynamics of COVID‐19 in Wuhan, Hao et al. (2020) estimate that the early stage *R_0_* of 3.54 has decreased to 0.28 as of March 8 corresponding to a reduction in total infections by 96%, shortly after the introduction of community‐based digital contact tracing. The eventually successful experience in Wuhan is broadly applied in many other Chinese cities that encountered the threat of a ‘second wave’. In China, several megacities underwent a similar process of community‐based digital contact tracing after the detection of local outbreaks including Harbin (April), Beijing (June), Dalian (July), Ürümqi (August), Qingdao (October), and Shenyang (December). In sum, the exploratory model of community‐based digital contact tracing has reached national adaptability and provides policymakers and ICT enterprises with a practical reference.

### 4.2 Ethical decision making

While the case of Wuhan largely emphasizes the effectiveness of community‐based digital contact tracing in curbing the spread of the virus as the priority, numerous crucial ethical issues are not yet sufficiently addressed in practice. First of all, the big data analytics of public health information at the social level raise enormous concerns on users’ informed consent, privacy, anonymization, data ownership, and right of access to data (Mittelstadt and Floridi, 2016). The lack of a reliable regulatory backbone on public data collection and analytics also weakens the legitimacy and credibility of the contact tracing measures introduced by the local governments. The digital platform governance in China is largely dependent on the self‐regulation of internet users under the guidance and trust‐building of the government (Weber and Jia, 2007). However, the Chinese authorities are broadly criticized for the actual misuse of sensitive personal data considering its strong data collection capability and ambiguous personal information standards (Greenleaf and Livingston, 2017). The terms of service of Wuhan’s contact tracing apps explicitly state that data encryption and masking processing are applied to protect users’ personal information. However, such general statements still cannot completely ease the privacy concerns of users without more transparent clarification. Individuals in the underground can hardly request the ICT firms or the government to disclose how sensitive personal data collected during digital contact tracing are stored and proceeded after the pandemic (Kelion, 2020).

Second, digitized public administration demands on the resolution of privacy concerns to enhance the willingness of citizen participation (Angst and Agarwal, 2009). Nonetheless, constrained by the information asymmetry in an ecosystem dominated by the government and enterprises, the general public’s willingness to provide information and the ‘right to know’ is often ignored or undermined by policymakers and enterprises (Dawson et al., 2010). In China, individual participation in digital contact tracing is mostly based on enforced obligation rather than voluntary motivation. For example, the contact tracers have the right to refuse individuals without valid health QR codes exhibited on the contact tracing apps to enter the community or public places and offices. In other words, the individuals have little freedom of choice even if they are not willing to participate in digital contact tracing.

Third, the community‐based digital contact tracing applied in Wuhan mostly emphasizes the effectiveness of bottom‐up data collection and analysis from the underground citizens, while the top‐down feedback and direction from the upperground organizations largely depend on the goodwill of the government. Local communities are regarded more as a grassroots unit of public administration mandated by the municipal government, rather than spontaneously formed autonomous collectives of residents. Whether the voice of the underground individual can be heard and transferred toward the upperground via the middleground community mechanisms remains unclear in China. Without accountable legislation and transparent monitoring by the middleground communities, upperground government, and tech giants monopolize the use of public data with little collective social forces to advocate the rights of the weaker side of the underground individuals.

## 5 Conclusion

In this article, we study how community‐based digital contact tracing contributes to curbing the spread of COVID‐19. The community‐based digital contact tracing incorporates enterprise innovation, citizen participation, and government decision making in the open innovation ecosystem and, in turn, contributes to the completeness and timeliness of big data analytics to contain the viral spread (Chun et al., 2010; Greve, 2015; Kretzschmar et al., 2020). As part of digital solutions to curb viral transmission, the digital contact tracing system developed by ICT firms provides real‐time public health information recording the geospatial data of diagnosed or suspected infections (Kamel Boulos and Geraghty, 2020). Compared to traditional manual contact tracing constrained by available human resources, scalable digital resources ‘would be sufficient to stop the epidemic if used by enough people, in particular when combined with other measures such as physical distancing’ (Ferretti et al., 2020). By targeting only those citizens at risk, epidemics could be contained without the need for mass quarantines (‘lockdowns’) that are harmful to society in numerous ways.

In addition to the technological inputs of enterprises, the effectiveness of such a digital solution also requires the synergy of governmental enforcement and guidelines as well as active citizen participation. The effectiveness of digital contact tracing depends on broad bottom‐up social engagement and the timely top‐down intervention, whereas context‐dependent social, technological, and political factors, including compliance with regulations, may moderate the outcomes (Braithwaite et al., 2020). Under the legal endorsement of the government and technological support of ICT firms, the digital contact tracing systems orchestrate the synergy of key stakeholders in an open innovation ecosystem. In this progress, local communities play the pivotal role of the middleground that stimulates the underground citizen participation and facilitates the upperground organizational decision making (Cohendet et al., 2010). Facilitated by the innovation outputs by the enterprises, communities are enabled to co‐produce policy with the government and deliver services to the public (Bovaird, 2007). As a result, the digital solutions ease the pressure on healthcare and public administrative systems, while maintaining a higher socioeconomic equilibrium (Ting et al., 2020).

As evidence, we explore the application of community‐based digital contact tracing in Wuhan as the prominent example of China’s ‘virus exceptionalism’. Since the beginning of the COVID‐19 pandemic, around 50 countries have launched government‐backed contact tracing apps: 80% are Bluetooth‐based and 90% function voluntarily (O'Neill et al., 2020). However, without government enforcement and community coordination, many of these Bluetooth‐based apps cannot reach sufficiently broad coverage of citizens or construct effective contact tracing systems for big data analytics based on geographic location and social relationships. The example of Wuhan illustrates how big data analytics on digital platforms are accelerated by broad social engagement and, in turn, contribute to re‐establishing socioeconomic order (Hao et al., 2020). Although China’s ‘virus exceptionalism’ may not be duplicable in most other countries, the general and China‐specific epidemiological evidence is confirmative of the positive influence of digital contact tracing in curbing the viral spread and complements the implications derived from the case study of Wuhan, which has strong theoretical and practical implications for further research (Ferretti et al., 2020; Hao et al., 2020; Kretzschmar et al., 2020). Meanwhile, we underline critical ethical concerns related to the protection of individual privacy, the transparency of public consent, and the accountability of public interest. The means of digitalization itself cannot fully solve all the aforementioned problems alone. The enforcement of privacy law, the improvement of transparency of public decision making, and the trust‐building of the government are all at stake for countries that implement community‐based digital contact tracing measures. The open exchange of best practices and experiences will be conducive to develop the most suitable albeit potentially diverse solutions based on political, social, technological, and economic idiosyncrasies.

We acknowledge several limitations of our research. Although the pragmatic research approach contributes new and timely insights on the factual details of the case of Wuhan, more rigorous positivist research is needed to better understand the causal effect between the community engagement in digital contact tracing and viral spread control effectiveness. Due to the still evolving situation of COVID‐19 in China and elsewhere, the eventual effectiveness of community‐based contact tracing requires a comprehensive empirical assessment after the pandemic. Finally, we call for future research on how policymakers may balance the technological effectiveness, public engagement, and ethical responsibility of digital contact tracing in different countries.

## Supporting information

Supplementary Material

## Notes

^1^
The average number of people who will catch a disease from one contagious person in a population that was previously free of infection and not vaccinated. A reproduction rate below 1 is the necessary condition to contain the viral spread (Braithwaite et al., 2020).

^2^
Among the world’s largest economies, China is expected to be the only one to achieve positive GDP growth (around 1.9%) in 2020 (IMF World Economic Outlook Database, 2020).

^3^
The recent review by Braithwaite et al. (2020) presents 15 high‐quality studies published in English, of which seven are designated to COVID‐19.

^4^
The Online Appendix lists nonscientific secondary sources accessed in the study.

^5^
The status reports are originally provided to the German Federal Foreign Office and cover the time period January 22 to March 20 2020. Upon journalist request and in accordance with the German Freedom of Information Act, such reports were publicly disclosed on November 9 and subsequently obtained by the authors.

^6^
Unless specified, all dates mentioned hereafter are designated to year 2020.

^7^
As of March 2020, WeChat has 983.18 million active users and Alipay has 698.56 million active users in China.
