# Supplementary material for: Decoding China’s COVID‐19 ‘virus exceptionalism’: Community‐based digital contact tracing in Wuhan
Source: R&D Management. 2021 Mar 23;51(4):339–51. doi: 10.1111/radm.12464 (PMC8251302; doi:10.1111/radm.12464)
Supplement: Supplementary file 1 — Supplementary Material [file RADM-51-339-s001.pdf]

Online Appendix (not for print)

- Wuhan Municipal Health Commission: Notification of Wuhan Municipal Health Commission on the current situation of pneumonia in city (in Chinese, 武汉市卫健委关于当前我市肺炎疫情的情况通报), [December 31, 2019]: http://wjw.wuhan.gov.cn/gsgg/202004/t20200430_1199576.shtml

- World Health Organization (WHO): Pneumonia of unknown cause – China, [January 5, 2020]: https://www.who.int/csr/don/05-january-2020-pneumonia-of-unkown-cause-china/en/

- Wuhan Municipal Health Commission: Experts interpret the latest notification of unexplained viral pneumonia (in Chinese, 专家解读不明原因的病毒性肺炎最新通报), [January 11, 2020]: http://wjw.wuhan.gov.cn/xwzx_28/gsgg/202004/t20200430_1199594.shtml

- National Health Commission of the People's Republic of China: The response of high-level expert group of the National Health Commission to journalists' questions on the pneumonia epidemic caused by the new coronavirus (in Chinese, 国家卫生健康委员会高级别专家组就新型冠状病毒感染的肺炎疫情答记者问), [January 21, 2020]: http://www.nhc.gov.cn/xcs/s7847/202001/8d735f0bb50b45af928d9944d16950c8.shtml

- Wuhan Municipal Health Commission: Headquarter for the Prevention and Control of Pneumonia Outbreak of Novel Coronavirus Infection of Wuhan: The No. 1 "Lockdown Order" issued by Wuhan Covid-19 Prevention and Control Command Department (in Chinese, 武汉市新型冠状病毒感染的肺炎疫情防控指挥部通告（第 1 号）), [January 23, 2020]: http://www.gov.cn/xinwen/2020-01/23/content_5471751.htm

- The State Council of the People's Republic of China: Position Adjustment of major leading members of the Hubei Provincial Party Committee (in Chinese, 湖北省委主要负责同志职务调整), [February 13, 2020]: http://www.gov.cn/xinwen/2020-02/13/content_5478048.htm#:~:text=%E6%96%B0%E5%8D%8E%E7%A4%BE%E5%8C%97%E4%BA%AC2%E6%9C%88,%E4%B9%A6%E8%AE%B0%E3%80%81%E5%B8%B8%E5%A7%94%E3%80%81%E5%A7%94%E5%91%98%E8%81%8C%E5%8A%A1%E3%80%82

- Epidemic Prevention and Control Headquarter of Wuhan: "Wuhan Health Code" updated online, citizens can scan the code to register for special reasons when going

out (in Chinese, 武汉：上线"武汉健康码"市民特殊原因外出可扫码登记), [February 22, 2020]: http://www.gov.cn/xinwen/2020-02/22/content_5482162.htm

- Hubei Provincial People's Government: What is the difference between the National Health Code, Hubei Health Code and Wuhan Health Code? Can information be consolidated? Please see the authoritative answer (in Chinese, 全国健康码、湖北健康码、武汉健康码有何区别？信息可否互通？请看权威答疑), [March 1, 2020]: https://www.hubei.gov.cn/zhuanti/2020/gzxxgzbd/hy/202003/t20200301_2165179.shtml

- Health Commission of Hubei Province: How was the Wuhan epidemic-free community assessed? (in Chinese, 武汉无疫情小区是怎么评出来的?), [March 7, 2020]: http://wjw.hubei.gov.cn/bmdt/ztzl/fkxxgzbdgrfyyq/fkdt/202003/t20200307_2174800.shtml

- Hubei Provincial People's Government: At 0:00 am today, Wuhan lifts Lockdown (in Chinese, 今日零时，武汉"解封"), [April 8, 2020]: http://www.hubei.gov.cn/2019/tpyw/202004/t20200408_2207205.shtml

- Wuhan Covid-19 epidemic prevention and control headquarter Full text of Wuhan's notification on revising numbers of confirmed COVID-19 cases, deaths, [April 17, 2020]: http://www.china.org.cn/china/Off_the_Wire/2020-04/17/content_75943843.htm

- Times: What Is Contact Tracing? Here's How It Could Be Used to Help Fight Coronavirus, [April 22,2020]: https://time.com/5825140/what-is-contact-tracing-coronavirus/

- The Economist: China Plans to Crush New Covid Outbreaks with Tough Measures, [April 30 2020]: https://www.economist.com/china/2020/04/30/china-plans-to-crush-new-covid-outbreaks-with-tough-measures

- The Economist: The pieces of the puzzle of Covid-19's origin are coming to light, [May 2, 2020]: https://www.economist.com/science-and-technology/2020/05/02/the-pieces-of-the-puzzle-of-covid-19s-origin-are-coming-to-light

- Press Conference of the Joint Prevention and Control Mechanisms of the State Council (in Chinese, 国务院联防联控机制新闻发布会), [May 7, 2020]：http://www.gov.cn/xinwen/gwylflkjz115/index.htm

- O'Neill, P. H., Ryan-Mosley, T. & Johnson, B. 2020. How to Submit a Change to the Covid Tracing Tracker Project. MIT Technology Review, [May 7 2020]:

https://www.technologyreview.com/2020/05/07/1001354/how-to-submit-a-change-to-the-covid-tracing-tracker-project

- Xinhua Press: Sudden increase of 6 diagnosed cases tracing several days after "Zeroization" in Wuhan: (in Chinese, 武汉"清零"多日后突增 6 名确诊病例追踪), [May 12, 2020]: http://www.xinhuanet.com/2020-05/12/c_1125976087.htm

- Wuhan Municipal Health Commission: Ten Q&A on centralized nucleic acid testing in Wuhan (in Chinese, 武汉市集中核酸检测十问十答), [May 16, 2020]: http://wjw.wuhan.gov.cn/ztzl_28/fk/tzgg/202005/t20200516_1321675.shtml

- Government of Hubei Province, 104th press conference of on prevention and control of Covid-19 (in Chinese, 新型冠状病毒感染的肺炎疫情防控工作"新闻发布会第 104 场), [June 2, 2020]: https://www.hubei.gov.cn/hbfb/xwfbh/202006/t20200602_2376181.shtml

- Beijing Municipal Health Commission, Clearance of all hospitalization of Xinfadi cluster of epidemic cases in Beijing (in Chinese, 北京新发地聚集性疫情 在院病例全部清零), [August 7,2020]: http://wjw.beijing.gov.cn/xwzx_20031/xwfb/202008/t20200807_1975976.html

- The National People's Congress of the People's Republic of China: Launch of the Personal Information Protection Law (Draft), October 15, 2020 (in Chinese, 个人信息保护法草案首次亮相), [October 15, 2020]: http://www.npc.gov.cn/npc/c30834/202010/569490b5b76a49c292e64c416da8c994.shtml

- The Economist: In a world mired in recession, China manages a V-shaped recovery, [October 22, 2020]: https://www.economist.com/china/2020/10/22/in-a-world-mired-in-recession-china-manages-a-v-shaped-recovery

- Statista: Leading apps by monthly active user number in China as of March 2020, Statista, [November 2020]: https://www.statista.com/statistics/1032630/china-leading-apps-by-monthly-active-users/
